# Prevalence of lower second molar impaction on panoramic radiographs of Peruvian individuals. A cross-sectional study

**DOI:** 10.4317/jced.63125

**Published:** 2025-10-01

**Authors:** Milagros Carina Rojas-Yauri, Carlos Jherson Arias-Quispe, Luis Ernesto Arriola-Guillén

**Affiliations:** 1 Undergraduate student, School of Dentistry, Universidad Científica del Sur, Lima, Perú; 2 Ph.D. and Associate Professor of the Division of Orthodontics, School of Dentistry, Universidad Científica del Sur, Lima, Perú

## Abstract

**Background:**

To determine the prevalence and primary radiographic characteristics of impacted mandibular second molars in Peruvian individuals using panoramic radiographs.

Material and methods: This cross-sectional study examined 1,000 digital panoramic radiographs of young adults aged 15 to 40 years in Lima, Peru, conducted from 2022 to 2024. These radiographs were evaluated individually for each side, for a total of 2000 sides evaluated. Two trained evaluators performed the measurements. The selected radiographs included complete lower dentition and demonstrated good contrast and clarity. We evaluated the presence of second molar impaction and the level of impaction (coronal, cervical, or radicular). The direction of impaction was also assessed and categorized as vertical, mesioangular, distoangular, horizontal, inverted, or transverse. Additionally, the relationship between the third molar and whether the impaction was unilateral (right or left) or bilateral were evaluated. Data analysis was performed using IBM SPSS Statistics version 29.0, applying Fisher’s exact test, chi-square test, and logistic regression (*p*<0.05).

**Results:**

The sample consisted of 514 females (mean age, 26.79 ± 10.12 years) and 486 males (mean age, 26.26 ± 9.76 years). The prevalence of impaction was found to be 6%. Among the impacted cases, females represented the majority (61.67%) compared to males (28.33%). The most frequently observed level of impaction was coronal (78.8%), followed by cervical (16.5%) and radicular (4.7%). The predominant angulation was mesioangular (71.8%), followed by distoangular (16.5%), vertical (7.1%), and horizontal (4.7%). Unilateral impaction was fairly distributed between the right side (28.3%) and left side (30%), while bilateral impaction was observed in 41.7% of cases.

**Conclusions:**

The prevalence of impacted second molars in our study was significant and has a considerable impact on orthodontic clinical practice. This issue was more common in females, with most cases occurring at the coronal level. The most frequent orientation of impaction was mesioangular, and unilateral cases were more common than bilateral ones.

** Key words:**Prevalence, Mandible, Tooth eruption, Panoramic radiograph, Molar tooth.

## Introduction

Tooth eruption is a dynamic process where a tooth moves through the alveolar bone from its initial position until it meets the opposing tooth. During this process, variations can occur due to genetic, systemic, and local factors [1-3]. Few studies indicate that changes in the eruption of mandibular second molars are relatively rare, affecting approximately 0.03% to 5.41% of the population [3-7]. These changes are often associated with factors such as improper positioning of tooth buds, blockages in the eruption path, and issues during the eruption process [2-5]. Typically, it presents with a mesial inclination, and there is a slight preference for females among those affected [5-7]. Also, this issue tends to occur more frequently on one side, particularly in the fourth quadrant of the mandible. However, this final aspect is not very clear.

Impaction refers to the failure of a tooth to erupt due to a physical obstacle in the eruption process. Impacted second molars are among the most common eruption problems associated with mandibular molars, second only to impacted third molars [7-9]. Several systemic and local factors contribute to this condition, including the distance between the mandibular first molar and the internal ramus of the mandible, the early eruption of the third molar, crowding in the posterior region, and an unusual inclination of the germ of the mandibular second molar [10-12]. Other studies show the ectopic position and failures in the eruption mechanism as frequent causes for the development of this type of impaction [13-15].

Panoramic radiography is the most effective method for identifying impacted molars, as it provides a clear view of root formation. A tooth that has developed more than three-quarters of its total root length but has not yet erupted is in questionable status [3]. However, if the direction of eruption is unfavorable or there is a reduced mental gonial angle, impaction is likely to occur [16]. In clinical practice, dentists frequently encounter impacted second molars, which can affect the eruption of adjacent third molars [17]. Delayed intervention for these impactions may lead to complications, such as root caries in the first molars, along with various periodontal, endodontic, and occlusal issues [8,9]. Therefore, early intervention is crucial; without it, the prognosis for these cases can be unfavorable.

Among the alternative solutions for impacted second molars, we have the elimination of obstacles such as dense fibrotic tissues that prevent normal eruption [16], extraction the usually impacted third molar and then verticalize the second molars, thus allowing for a more accepTable occlusion [10,11], extraction of the second molar and mesialization of the third molar, although there is a risk of root resorption. [18,19]. Generally, the more developed the root of the tooth, the more challenging it becomes to correct, highlighting the importance of early intervention as the most effective approach [16,20]. In adults, verticalization of impacted second molars is typically the first treatment option since extracting this tooth could result in occlusal changes, such as the extrusion of opposing teeth [10,12].

Research on the prevalence of impacted second molars is limited [3-5], with only a few studies available. Further research is needed to enable clinicians to manage these challenging cases effectively. Therefore, this study aims to determine the prevalence and key imaging characteristics of impacted mandibular second molars in Peruvian individuals using panoramic radiographs.

## Material and Methods

The study utilized a descriptive, observational, cross-sectional, and retrospective design. The Institutional Ethics Committee for Research at Universidad Científica del Sur approved the study under code 862-CIEI-CIENTÍFICA-2025.

The study used a database of panoramic radiographs from individuals who visited a private radiological center in Lima, Peru, between 2022 and 2024. To achieve access to this database, the researchers submitted a formal request to the center, which was approved after a few weeks. The request detailed the purpose of the study and asked for authorization to access the information contained in their records. Once permission was granted and the necessary report was provided, the researchers reviewed the digital panoramic radiographs. They secured a backup of the sample to ensure the integrity of the information.

Using the Fisterra software, a minimum sample size of 203 panoramic radiographs was calculated using a formula for estimating a proportion in an infinite population. This estimate was based on a 95% confidence level, a 3% precision rate, and an expected proportion of 5%. However, 1,000 radiographs were included in the study, selected through simple random sampling. Each side of the radiographs was evaluated individually, totaling 2000 sides assessed.

The inclusion criteria for the study were panoramic radiographs that exhibited good contrast and sharpness, featuring young adults aged 15 to 40 years of both sexes who had all their permanent mandibular teeth. Radiographs were excluded if they belonged to individuals with a history of extraction of permanent mandibular teeth, previous mandibular surgical procedures, or the presence of fixed orthodontic appliances in the molar area near the impaction.

A pilot test was conducted using 250 panoramic radiographs, which included 104 from male individuals and 146 from female individuals. Two researchers received training and calibration, with their evaluations supervised by an orthodontist with over 10 years of experience in the field. They measured all the variables for this study. The inter-observer agreement was assessed using Cohen’s Kappa index, resulting in a value of 1, indicating almost perfect agreement.

Data collection was conducted using digital tracings in Microsoft PowerPoint, employing a direct observation method. Two trained and calibrated researchers assessed the presence of second molar impaction, defined as an abnormal eruption direction of a fully formed second molar that has not emerged through the gums due to obstruction. We measured the level of impaction, determined by the depth of the lower second molar about the occlusal plane of the lower first molar, using the Pell and Gregory classification [13]. This classification categorizes impaction into three levels: coronal, cervical, and radicular. The direction of the impaction was defined by the orientation of the long axis of the lower second molar concerning the long axis of the lower first molar and evaluated according to Winter’s classification. The recorded positions included vertical, mesioangular, distoangular, horizontal, inverted, and transverse. We also examined the relationship or contact with the third molar. Lastly, we assessed the condition of the impaction, specifically whether it was unilateral on the right side, unilateral on the left side, or bilateral. Additionally, we collected demographic data, such as sex and age.

- Statistical analysis

The collected data was processed and analyzed using IBM SPSS Statistics for Windows, version 29.0 (IBM Corp., Armonk, NY, USA). We conducted Fisher’s exact test, chi-square tests, and logistic regression analysis to evaluate the factors influencing the frequency of impactions in permanent mandibular second molars. This analysis examined variables such as sex, level, direction, and condition of the second molar impaction, as well as the relationship with the third molar. A significant level of *p* < 0.05 was used for all tests.

## Results

In this study, we reviewed a total of 1,000 panoramic radiographs from individuals aged 15 to 40 years, assessing a total of 2,000 sides. Among these, 486 radiographs (mean age 26.26 ± 9.76 years) were from females, and 514 radiographs (mean age 26.79 ± 10.12 years) were from males. A statistically significant difference was observed between the two groups (*p* = 0.030, see  Table 1).

The prevalence of impacted mandibular second molars was found to be 6% (n = 60), while 94% (n = 940) showed no signs of impaction. When analyzing the distribution by sex, females had a slightly higher and statistically significant rate of impaction (7.2%, *n* = 37) compared to males (4.7%, *n* = 23), with a *p-value* of 0.024 ( Table 2). Among the 60 panoramic radiographs depicting impacted molars, a total of 85 impacted mandibular second molars were identified when analyzed individually by side. The evaluation of the level of impaction by sex revealed that the majority of cases were classified as coronal impactions (78.8%, *n* = 67), followed by cervical (16.5%, *n* = 14) and radicular (4.7%, *n* = 4) impactions. However, these differences were not statistically significant (*p* = 0.240,  Table 3).

Regarding the direction of impaction by sex, the most frequently observed type was mesioangular (71.8%, *n* = 61), followed by distoangular (16.5%, *n* = 14), vertical (7.1%, *n* = 6), and horizontal (4.7%, *n* = 4). No cases of inverted or transverse impaction were identified ( Table 4).

To assess the association with the presence of impacted mandibular third molars, each side of the 1,000 panoramic radiographs was evaluated, resulting in a total of 2,000 sides analyzed. Among these, 65.1% (n = 1,303) exhibited impacted third molars, while 34.8% (n = 697) showed no impact. This difference was not statistically significant (*p* = 0.060,  Table 5). Among the 60 panoramic radiographs evaluated for impaction, 41.7% (n = 25) showed bilateral impaction, 30% (n = 18) were impacted on the left side, and 28.3% (n = 17) were impacted on the right side (*p* = 0.080,  Table 6).

Finally, binary logistic regression analysis revealed that the interaction between age and sex in the occurrence of impacted second molars was statistically significant, with sex appearing to have a greater influence on females (OR = 1.566; 95% CI, as shown in  Table 7).

## Discussion

Mandibular second molar impaction is a rare developmental dental anomaly characterized by the improper eruption of the tooth, often caused by physical or anatomical obstructions along its eruption path [1-8]. This condition can lead to changes in occlusion, necessitating treatment. However, how common is this issue? This research aims to determine the prevalence and primary radiographic features of a mandibular second molar impaction as observed in panoramic radiographs of individuals in Peru.

In the present study, we found a prevalence rate of 6%, which is higher than the rates reported in most previous studies, where prevalence ranged from 0.13% to 5.41% [3-5] However, this finding is comparable to the results of David *et al*. [5], who reported a prevalence of 5.41% in a sample of 1,756 individuals in Colombia. The differences in prevalence rates among these studies may be due to various methodological or demographic factors, such as age range, genetic differences, or inclusion criteria. In our study, we selected only panoramic radiographs that included all mandibular teeth, which may have improved diagnostic accuracy. However, this approach might also limit the inclusion of cases with agenesis or missing teeth, thereby reducing the observed prevalence.

Regarding sex, among the 60 panoramic radiographs, a higher frequency of impaction was found in females (61.67%) compared to males (33.33%), a statistically significant difference (*p* = 0.024). This association was confirmed through the binary logistic regression model, in which female sex was a significant predictor of impaction (*p* = 0.020). Females were found to be 1.57 times more likely to develop impaction compared to males (OR = 1.566; 95% CI). This finding aligns with that of David *et al*. [5], who observed a higher prevalence in females (60.27%) compared to males (39.73%). Similarly, Fan *et al*. [6] found a distribution of 63.93% in females and 36.07% in males, suggesting a possible female predisposition to this condition. However, Kuang *et al*. [4] found no significant differences between the sexes (*p* = 0.081), reporting a distribution of 59.8% in males and 40.2% in females.

The level of impaction was mainly located at the coronal level (78.8%), followed by the cervical level (16.5%) and, to a lesser extent, the radicular level (4.7%). The direction of impaction was predominantly mesioangular (71.8%), followed by distoangular (16.5%), vertical (7.1%), and horizontal (4.7%). These results are consistent with those reported by Kuang *et al*. [4], who found 74.45% mesioangular impactions, 13.87% horizontal, 4.38% vertical, and 5.11% distoangular. Similarly, Fan *et al*. [6] reported a distribution of 73.6% mesioangular, 14.2% horizontal, and 12.2% vertical. David *et al*. [5] also reported a predominantly mesial angulation (96.8%). The consistency of these findings across different populations reinforces the notion that mesioangular angulation is the most common pattern of mandibular second molar impaction.

In our study examining the association with third molars, 2,000 mandibular sides were evaluated, revealing that impacted third molars were present in 65.1% of cases, while 34.8% exhibited no impaction. This high coexistence rate suggests a direct relationship between the occlusal eruption of second and third molars, emphasizing the importance of comprehensive evaluations in radiographic diagnosis.

Regarding the condition of impaction, the distribution was as follows: 41.7% of cases were bilateral, 30% were on the left side, and 28.3% were on the right side, resulting in a total of 58.3% unilateral cases. These findings are somewhat similar to those reported by Kuang *et al*. [4], who found a predominance of unilateral impactions (72.3%) and a lower frequency of bilateral cases (27.7%) in a sample of 112 Chinese patients. In contrast, David *et al*. [5] reported a higher proportion of bilateral cases (58.9%), with 29.5% on the left side and 11.6% on the right side. The differences observed may be attributed to genetic factors, variations in mandibular development between Asian and Latin American populations, or discrepancies in diagnostic and inclusion criteria across studies.

A limitation of this study is that it was a retrospective investigation based solely on panoramic radiographs from patients treated at a single private radiological center in Lima. This narrow focus may limit the generalizability of the results to other populations. Furthermore, the study lacked complementary clinical information, such as medical histories and oral habits, which hindered the analysis of additional potential etiological factors. Future research could investigate these variables to enrich the findings in this field further.

Overall, the results of this study align with recent scientific literature and provide updated epidemiological evidence on a relatively rare but clinically significant condition. These findings underscore the importance of timely radiographic evaluations, which enable the early detection of mandibular second molar impaction, thereby facilitating the planning of preventive or therapeutic interventions.

## Conclusions

The findings of this study indicate a significant prevalence of impacted permanent mandibular second molars within the analyzed population, surpassing the rates typically reported in international research. A higher incidence of impaction was noted in females. The most common level of impaction was coronal, and the prevailing angulation was mesioangular. Additionally, the distribution of impaction cases was relatively balanced between unilateral (left and right) and bilateral cases, although there was a slight predominance of bilateral cases.

## Figures and Tables

**Table 1:** Initial characteristics of the sample according to age and sex.

Sex	n	Media	SD
Male	486	25.26	9.76
Female	514	26.79	10.12

*p*=0.230* Students’ T-Test *Significant

**Table 2:** Prevalence of second molar impaction according to sex.

Sex		Second molar impaction		Total
		Absent	Present	
Male	n	463	23	486
	%	95.3	4.7	100
Female	n	477	37	514
	%	92.8	7.2	100
Total	n	940	60	1000
	%	94	6	100

*p*=0.024* Fisher’s exact test *Significant

**Table 3:** Level of second molar impaction according to sex.

Sex		Level of impaction			Total
		Coronal	Cervical	Radicular	
Male	n	23	6	3	32
	%	71.9	18.8	9.4	100
Female	n	44	8	1	53
	%	83	15.1	1.9	100
Total	n	67	14	4	85
	%	78.8	16.5	4.7	100

*p*=0.240* Chi-square test *Not significant

**Table 4:** Direction of second molar impaction according to sex.

Sex		Direction of impaction				Total
		Vertical	Mesioangular	Distoangular	Horizontal	
Male	n	4	12	12	4	32
	%	12.5	37.5	37.5	12.5	100
Female	n	2	49	2	0	53
	%	3.8	92.5	3,8	0	100
Total	n	6	61	14	4	85
	%	7.1	71.8	16.5	4.7	100

*p* <0.001* Chi-square test *Significant

**Table 5:** Relationship of second molar impaction with the third molar, according to sex.

Sex		Relationship with the third molar		Total
		Absent	Present	
Male	n	359	613	972
	%	36,9	63,1	100
Female	n	338	690	1028
	%	32.9	67.1	100
Total	n	697	1303	2000
	%	34,8	65,1	100

*p*=0.060 Chi-square test

**Table 6:** Table Side of second molar impaction according to sex.

Sex		Side of impaction			Total
		Right	Left	Bilateral	
Male	n	10	4	9	23
	%	43.5	17.4	39.1	100
Female	n	7	14	16	37
	%	18.9	37.8	43.2	100
Total	n	17	18	25	60
	%	28.3	30	41.7	100

*p*=0.080 Chi-square test

**Table 7:** Table Binary logistic regression to determine the influence of sex and age on the occurrence of impacted second molars.

Variables	p	Exp. (B)	95% C.I. for EXP(B)	
			Lower	Upper
Male	---	---	---	---
Female	0.020	1.566	1.072	2.288
Age	0.596	0.995	0.976	1.014
Constant	<0.001*	0.057		

*Significant

## Data Availability

The datasets used and/or analyzed during the current study are available from the corresponding author.
